# The relationship between dietary vitamin B1 and stroke: a machine learning analysis of NHANES data

**DOI:** 10.3389/fnut.2025.1584654

**Published:** 2025-05-06

**Authors:** Shihan Guo, Xu Jiao, Mingfei Li, Zhuo Li, Yun Lu

**Affiliations:** ^1^Emergency Department, Hospital of Chengdu University of Traditional Chinese Medicine, Chengdu, China; ^2^Clinical Medical School, Chengdu University of Traditional Chinese Medicine, Chengdu, China; ^3^Department of Nursing, Tongji Hospital, Tongji Medical College, Huazhong University of Science and Technology, Wuhan, China

**Keywords:** vitamin B1, stroke, cross-sectional study, machine learning, NHANES

## Abstract

**Background:**

Vitamin B1 deficiency is closely linked to damage in the cardiovascular system. However, the relationship between dietary Vitamin B1 intake and the risk of stroke remains ambiguous and requires further investigation.

**Methods:**

This study analyzed data from participants in the National Health and Nutrition Examination Survey (NHANES: 2005–2018) to investigate the relationship between dietary vitamin B1 and ischemic stroke. Weighted multivariable logistic regression models and restricted cubic spline (RCS) regression were employed to explore potential nonlinear relationships, and subgroup analyses were conducted to assess the robustness of the results. Additionally, the Least Absolute Shrinkage and Selection Operator (LASSO) was utilized for feature selection. Eight machine learning methods were employed to construct predictive models and evaluate their performance. Based on the best-performing model, we further examined variable importance and model accuracy, employing Shapley Additive Explanations (SHAP) analysis to interpret the model. Finally, a nomogram was created to enhance the readability of the predictive model results.

**Results:**

After controlling for various variables, vitamin B1 exhibited a significant linear negative correlation with stroke risk. In comparison to the lowest quartile, the adjusted odds ratio (OR) for the fourth quartile was notably reduced to 0.66 (95% CI: 0.46, 0.94). Restricted cubic spline (RCS) analysis further confirmed a linear inverse relationship between vitamin B1 levels and stroke risk. Moreover, the Gradient Boosting Machine (GBM) model demonstrated robust predictive efficacy, achieving an area under the curve (AUC) of 91.9%.

**Conclusion:**

A large-scale study based on NHANES indicates that as dietary intake of vitamin B1 increases, the risk of stroke shows a gradual decline. Therefore, appropriately increasing dietary intake of vitamin B1 may reduce the risk of stroke occurrence.

## 1 Introduction

Stroke, clinically referred to as a cerebrovascular accident, is a syndrome of brain dysfunction resulting from pathological events in the cerebral blood vessels, including arterial embolism, small vessel disease, or cerebral infarction. Clinically, it is primarily categorized into two types: hemorrhagic and ischemic, which account for 20 and 80% of all strokes, respectively (1). According to the World Health Organization (WHO), ~15 million individuals experience a stroke annually, with around 6 million of these cases resulting in death (1, 2). While the incidence of stroke typically increases with age, recent trends indicate a rising incidence among younger populations (3). The primary risk factors for stroke include hypertension, diabetes, high cholesterol, and smoking (4).

B vitamins play a unique and essential role in both the central nervous system (CNS) and the peripheral nervous system (PNS). Studies have demonstrated that a deficiency in B vitamins can result in elevated levels of homocysteine in the blood, and high homocysteine levels are associated with an increased risk of stroke (5, 6). This may occur through mechanisms such as endothelial dysfunction, oxidative stress, and inflammatory responses (7). As a vital component of the B vitamin group, the active form of vitamin B1 is thiamine, which is involved in glucose metabolism, the maintenance of neural membrane function, and the synthesis of myelin and several neurotransmitters, including acetylcholine, serotonin, and amino acids (8, 9). Additionally, thiamine provides restorative capabilities, leading to the hypothesis that it may possess antioxidant effects, thereby protecting nerve cells (10, 11). This may be closely related to stroke prevention. Although some epidemiological studies have explored the relationship between B vitamins and stroke, the majority of research has concentrated on folic acid, niacin, vitamin B6, and vitamin B12 (12–14). However, the link between vitamin B1 and stroke remains largely unexplored.

This study aims to re-examine the relationship between vitamin B1 and stroke in the general population by analyzing data from the National Health and Nutrition Examination Survey (NHANES). We will employ advanced analytical methods to process the data and ensure its quality and reliability. Simultaneously, this study seeks to create and evaluate a proficient and interpretable machine learning (ML) system for predicting stroke risk. Our research findings offer a novel approach for the early identification of stroke and contribute to the advancement of ML in clinical research on brain diseases.

## 2 Methods

### 2.1 Study population

NHANES is conducted biennially by the Centers for Disease Control and Prevention (CDC) to assess the health and nutritional status of the U.S. population. This study utilized data from NHANES, provided by the National Center for Health Statistics (NCHS). NHANES is a comprehensive survey designed to collect representative information on the health and nutritional status of the civilian, non-institutionalized population in the United States, encompassing demographics, socioeconomic status, dietary habits, and health-related issues. To ensure sample diversity, NHANES employs a stratified, multistage probability sampling method to select nationally representative participants. The study protocol received approval from the CDC NCHS Ethics Review Board, and all participants provided written informed consent. The data are publicly accessible at https://www.cdc.gov/nchs/nhanes/.

**Figure 1:**
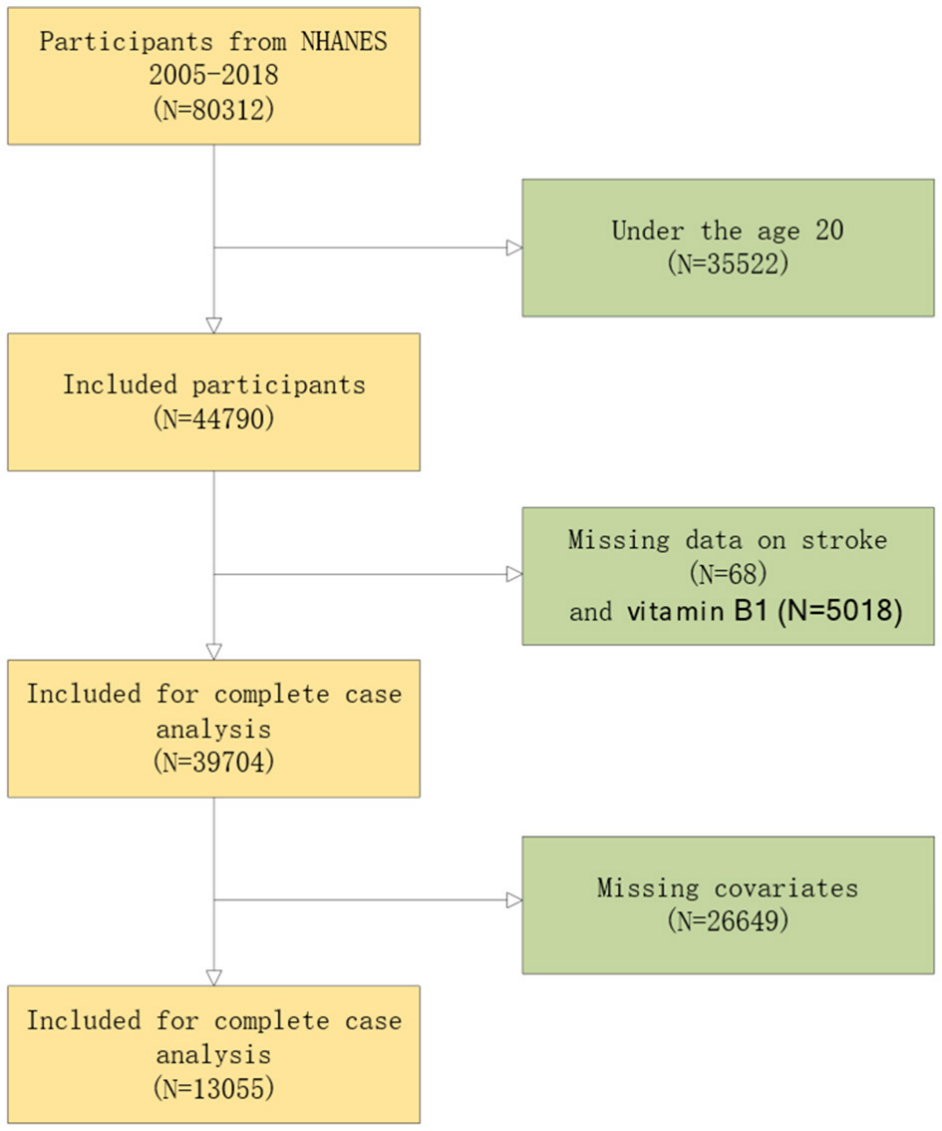
A detailed flow chart of participant recruitment.

This study primarily analyzed adult health data from NHANES 2005–2018. The original cohort included 80,312 participants. Initially, individuals under the age of 20 were excluded, followed by the exclusion of those lacking data on stroke, dietary vitamin B1, and related covariates. Ultimately, 13,055 participants were included in the final analysis. The sample selection flowchart is presented in Figure 1.

### 2.2 Dietary vitamin B1 intake

The NHANES study employed a 24-h dietary recall questionnaire, accessible to all participants, which facilitated the collection of comprehensive data regarding the types and quantities of foods consumed in the preceding 24 h. All NHANES participants were eligible for two 24-h dietary recall interviews, and the data collected were utilized to determine each individual's daily vitamin B1 intake. The initial dietary recall interview was conducted face-to-face at the Mobile Examination Center (MEC), while the second interview took place via telephone within a span of 3–10 days. Dietary vitamin B1 intake was assessed by calculating the average of the data obtained from the two 24-h dietary recalls. Participants were categorized based on their vitamin B1 intake.

### 2.3 Diagnosis of stroke

In this study, stroke identification relies on individuals disclosing previous diagnoses made by healthcare professionals during face-to-face interviews. Those who answered affirmatively to the question, “Has a doctor or healthcare provider ever told you that you had a stroke?” were classified as having a history of stroke. It is important to acknowledge that the use of self-reported data may be subject to memory bias, which could potentially affect the interpretation of the information (15). Although the NHANES database lacks specific details regarding the types of strokes, the relatively high prevalence of ischemic stroke among stroke patients suggests that a significant proportion of participants diagnosed with stroke may have experienced ischemic stroke.

### 2.4 Covariates

Demographic data were collected using a standardized survey that encompassed gender, race/ethnicity, educational background, smoking habits, alcohol consumption, poverty income ratio (PIR), body mass index (BMI), blood pressure (BP), total cholesterol (TC), LDL-C, and HDL-C. Alcohol consumption status was determined from two 24-h dietary recalls, with participants classified as alcohol users if they reported consuming alcohol in at least one of the dietary recalls (16). Smoking status was categorized into three groups: never smoker (<100 cigarettes), former smoker (individuals who are not currently smoking but have smoked 100 or more cigarettes), and current smoker (those who have smoked 100 or more cigarettes and currently smoke either daily or occasionally). Diabetes was defined based on the fulfillment of any of the following criteria: (1) HbA1c levels of 6.5% or greater; (2) fasting plasma glucose (FPG) levels of 7.0 mmol/L or higher; (3) random plasma glucose (RPG) levels of 11.1 mmol/L or above or an oral glucose tolerance test (OGTT) result of 11.1 mmol/L or higher; (4) a diagnosis of diabetes made by a physician; or (5) the current use of antidiabetic medication or insulin (17).

### 2.5 Statistical analysis

Due to the complex sampling methodology employed by the NHANES survey, our analytical approach incorporated sample weights specifically tailored for the study period to ensure accurate calculations of health-related statistics. These weights were adjusted for survey design, non-response, and post-stratification to ensure that the results are representative of the U.S. population. Weighted averages and 95% confidence intervals were utilized to represent the variables, ensuring that our estimates accurately reflect the population parameters.

Participants were divided into two groups based on the presence or absence of stroke and further categorized into four groups according to vitamin B1 quartiles. Continuous variables were assessed using the weighted Student's *t*-test or analysis of variance, while categorical variables were evaluated using the weighted chi-square test. For continuous variables that did not conform to a normal distribution, the weighted Kruskal-Wallis test was employed. In descriptive analyses, continuous variables were expressed as weighted means ± standard deviations, and categorical variables were reported as weighted percentages. To investigate the relationship between vitamin B1 and the risk of diabetes, we initially constructed three multivariate logistic regression models. Model 1 was unadjusted, while Model 2 was adjusted for gender, age, and race. Model 3 included further adjustments for education level, smoking status, alcohol consumption, body mass index (BMI), diabetes, and hypertension. To explore the potential nonlinear association between vitamin B1 scores and stroke, we employed restricted cubic spline (RCS) regression, with knots placed at the 5th, 35th, 65th, and 95th percentiles of the vitamin B1 score distribution. Additionally, subgroup analyses were conducted to examine whether significant interactions existed between these covariates and the association of vitamin B1 with stroke.

Traditional statistical methods are typically suitable for small datasets but struggle to interpret complex interaction patterns in high-dimensional data. Machine learning methods, on the other hand, are well-suited for large-scale data and can automatically adjust models to optimize performance through techniques such as parameter tuning and cross-validation (18, 19). This technology has been proven to perform well across multiple fields (20). In this study, we constructed a predictive model for stroke based on the variables included. We utilized univariate analysis and LASSO regression to identify the most significant characteristics associated with stroke risk. Subsequently, we employed eight machine learning (ML) algorithms: Logistic Regression (LR), Gradient Boosting Machine (GBM), Extreme Gradient Boosting (XGBoost), Support Vector Machine (SVM), Neural Network (NNET), Adaptive Boosting (AdaBoost), Light Gradient Boosting Machine (LightGBM), and Categorical Boosting (CatBoost) to train and develop ML models using a 10-fold cross-validation method.

The model's performance was evaluated using various metrics, including the area under the receiver operating characteristic curve (ROC), accuracy, sensitivity, specificity, F1 score, calibration curve, decision curve, clinical impact curve, and confusion matrix. Shapley Additive Explanations (SHAP) analysis was conducted to interpret the model, elucidating the importance of each feature and the rationale behind the model's decisions. Finally, a nomogram was constructed to enhance the interpretability of the predictive model results. Statistical analysis was conducted using R software version 4.4.2, with a two-sided *p*-value of < 0.05 considered statistically significant.

## 3 Results

### 3.1 Baseline characteristics of participants

This study included a total of 13,055 participants, comprising 12,556 non-stroke individuals and 499 stroke patients. Compared to the non-stroke group, stroke patients were predominantly female, over the age of 60, overweight, and had higher incidences of diabetes, hypertension, and smoking, along with lower educational attainment. These findings are consistent with common perceptions. Conversely, we observed that the incidence of stroke was lower among individuals who consumed alcohol and those with lower vitamin B1 intake. This discrepancy may be attributed to our definition of alcohol consumption, wherein individuals who consumed alcohol within 48 h, as recorded in the dietary data, were classified as drinkers. Further details can be found in Table 1.

**Table 1:** Baseline characteristics grouped by stroke.

**Characteristic**	**Overall, *N* = 13,055 (100%)^a^**	**Non- stroke, *N* = 12,556 (97%)^a^**	**Stroke, *N* = 499 (3.1%)^a^**	***P*-value^b^**
Age, years	27.00 (13.00, 41.00)	26.00 (13.00, 41.00)	34.00 (8.00, 51.00)	0.3
**Age, group**, ***n*** **(%)**				**<0.001**
40–60	4,361.00 (38.25%)	4,246.00 (38.56%)	115.00 (28.63%)	
≥60	4,354.00 (27.42%)	3,990.00 (26.20%)	364.00 (65.61%)	
20–40	4,340.00 (34.32%)	4,320.00 (35.24%)	20.00 (5.76%)	
**Sex**, ***n*** **(%)**				**0.032**
Female	6,812.00 (52.36%)	6,557.00 (52.12%)	255.00 (59.79%)	
Male	6,243.00 (47.64%)	5,999.00 (47.88%)	244.00 (40.21%)	
**Race**, ***n*** **(%)**				**0.008**
Non-Hispanic White	5,975.00 (69.92%)	5,716.00 (69.91%)	259.00 (70.09%)	
Non-Hispanic Black	2,647.00 (10.37%)	2,507.00 (10.21%)	140.00 (15.41%)	
Mexican American	1,960.00 (7.60%)	1,914.00 (7.68%)	46.00 (5.23%)	
Other	1,297.00 (7.04%)	1,271.00 (7.05%)	26.00 (6.84%)	
Other Hispanic	1,176.00 (5.07%)	1,148.00 (5.15%)	28.00 (2.43%)	
**BMI**, ***n*** **(%)**				**0.011**
Obese (30 or greater)	4,961.00 (36.71%)	4,730.00 (36.40%)	231.00 (46.44%)	
Overweight (25 to <30)	4,304.00 (32.76%)	4,155.00 (33.00%)	149.00 (25.47%)	
Normal (18.5 to <25)	3,589.00 (28.98%)	3,479.00 (29.09%)	110.00 (25.58%)	
Underweight (<18.5)	201.00 (1.55%)	192.00 (1.52%)	9.00 (2.50%)	
**Alcohol**, ***n*** **(%)**				**<0.001**
No	8,993.00 (64.47%)	8,576.00 (63.96%)	417.00 (80.54%)	
Yes	4,062.00 (35.53%)	3,980.00 (36.04%)	82.00 (19.46%)	
**Smoke**, ***n*** **(%)**				**<0.001**
Never	7,152.00 (53.57%)	6,956.00 (54.07%)	196.00 (37.99%)	
former	3,316.00 (26.36%)	3,133.00 (26.07%)	183.00 (35.29%)	
Current	2,587.00 (20.08%)	2,467.00 (19.86%)	120.00 (26.72%)	
**Educational**, ***n*** **(%)**				**<0.001**
≥High school	10,115.00 (85.24%)	9,794.00 (85.66%)	321.00 (71.94%)	
<High school	2,940.00 (14.76%)	2,762.00 (14.34%)	178.00 (28.06%)	
PIR	94.00 (1.00, 231.00)	94.00 (1.00, 231.00)	94.00 (24.00, 232.00)	0.2
**Hypertension**, ***n*** **(%)**				**<0.001**
No	8,302.00 (66.14%)	8,177.00 (67.31%)	125.00 (29.64%)	
Yes	4,753.00 (33.86%)	4,379.00 (32.69%)	374.00 (70.36%)	
**Diabetes**, ***n*** **(%)**				**<0.001**
No	10,729.00 (86.40%)	10,428.00 (87.08%)	301.00 (65.29%)	
Yes	2,326.00 (13.60%)	2,128.00 (12.92%)	198.00 (34.71%)	
TC, mg/dL	53.00 (25.00, 91.00)	53.00 (25.00, 91.00)	60.00 (22.00, 106.00)	0.2
HDL-C, mg/dL	19.00 (9.00, 33.00)	19.00 (9.00, 33.00)	23.00 (8.00, 38.00)	0.4
LDL-C, mg/dL	46.00 (21.00, 79.00)	46.00 (21.00, 78.00)	50.00 (23.00, 93.00)	0.069
WBC, × 109/L	22.00 (9.00, 40.00)	22.00 (9.00, 40.00)	23.00 (9.00, 45.00)	0.4
RBC, × 109/L	59.00 (26.00, 106.00)	59.00 (26.00, 106.00)	70.00 (29.00, 119.00)	**0.004**
PLT, × 106/L	77.00 (36.00, 135.00)	77.00 (36.00, 135.00)	78.00 (31.00, 147.00)	0.6
Vitamin B1, mg	1,261.00 (528.00, 2,470.00)	1,264.00 (534.00, 2,488.00)	1,059.00 (433.00, 2,080.00)	**<0.001**

a Median (IQR) for continuous; *n* (%) for categorical.

b Design-based Kruskal Wallis test; Pearson's X^∧^2: Rao & Scott adjustment CI, confidence interval; BMI, body mass index; PIR Ratio of family income to poverty; TG, triglycerides; LDL-C, low-density lipoprotein cholesterol; HDL-C, high-density lipoprotein cholesterol; RBC, red blood cells; WBC, white blood cells; PLT, platelets. Values in bold are statistically significant (*p* < 0.05).

To investigate the relationship between vitamin B1 and the risk of stroke, participants were divided into four quartiles (Q1-Q4) based on their vitamin B1 levels. The results indicated that participants in the higher quartiles of vitamin B1 exhibited significantly lower levels of total cholesterol (TC), platelet count (PLT), white blood cells (WBC), low-density lipoprotein cholesterol (LDL-C), and high-density lipoprotein cholesterol (HDL-C) compared to those in the lower quartiles (*p* < 0.05). Furthermore, significant differences were observed in the distribution of gender, race, education level, smoking status, and the prevalence of hypertension and diabetes (*p* < 0.05). Notably, as the quartiles of vitamin B1 increase, the prevalence of stroke significantly decreases (1.83 vs. 4.63%, *p* < 0.001). For further details, please refer to Table 2.

**Table 2:** Basic characteristics of participants by vitamin B1 quartile.

**Characteristic**	**Overall, *N* = 13,055 (100%)^a^**	**Q1, *N* = 3,584 (25%)^a^**	**Q2, *N* = 3,325 (25%)^a^**	**Q3, *N* = 3,147 (25%)^a^**	**Q4, *N* = 2,999 (25%)^a^**	***P*-value^b^**
Age, years	27.00 (13.00, 41.00)	27.00 (12.00, 42.00)	26.00 (12.00, 42.00)	27.00 (13.00, 42.00)	26.00 (13.00, 40.00)	0.5
**Age, group**, ***n*** **(%)**						**<0.001**
40–60	4,361.00 (38.25%)	1,148.00 (37.87%)	1,080.00 (36.27%)	1,082.00 (38.56%)	1,051.00 (40.32%)	
≥60	4,354.00 (27.42%)	1,351.00 (30.84%)	1,223.00 (31.14%)	1,017.00 (26.78%)	763.00 (20.92%)	
20–40	4,340.00 (34.32%)	1,085.00 (31.29%)	1,022.00 (32.59%)	1,048.00 (34.65%)	1,185.00 (38.76%)	
**Sex**, ***n*** **(%)**						**<0.001**
Female	6,812.00 (52.36%)	2,470.00 (71.77%)	1,969.00 (60.21%)	1,527.00 (49.23%)	846.00 (28.20%)	
Male	6,243.00 (47.64%)	1,114.00 (28.23%)	1,356.00 (39.79%)	1,620.00 (50.77%)	2,153.00 (71.80%)	
**Race**, ***n*** **(%)**						**<0.001**
Non-Hispanic White	5,975.00 (69.92%)	1,440.00 (64.75%)	1,522.00 (70.37%)	1,498.00 (71.14%)	1,515.00 (73.41%)	
Non-Hispanic Black	2,647.00 (10.37%)	948.00 (14.66%)	685.00 (10.36%)	572.00 (9.14%)	442.00 (7.32%)	
Mexican American	1,960.00 (7.60%)	530.00 (7.77%)	493.00 (7.11%)	449.00 (7.51%)	488.00 (8.01%)	
Other	1,297.00 (7.04%)	313.00 (7.13%)	332.00 (7.30%)	341.00 (7.28%)	311.00 (6.45%)	
Other Hispanic	1,176.00 (5.07%)	353.00 (5.68%)	293.00 (4.86%)	287.00 (4.93%)	243.00 (4.81%)	
**BMI**, ***n*** **(%)**						0.3
Obese (30 or greater)	4,961.00 (36.71%)	1,473.00 (39.20%)	1,305.00 (37.79%)	1,156.00 (35.43%)	1,027.00 (34.41%)	
Overweight (25 to <30)	4,304.00 (32.76%)	1,129.00 (30.98%)	1,095.00 (32.67%)	1,054.00 (33.45%)	1,026.00 (33.95%)	
Normal (18.5 to <25)	3,589.00 (28.98%)	919.00 (28.34%)	883.00 (28.27%)	886.00 (29.39%)	901.00 (29.91%)	
Underweight (<18.5)	201.00 (1.55%)	63.00 (1.49%)	42.00 (1.27%)	51.00 (1.72%)	45.00 (1.73%)	
**Alcohol**, ***n*** **(%)**						**<0.001**
No	8,993.00 (64.47%)	2,641.00 (69.41%)	2,311.00 (65.01%)	2,132.00 (64.03%)	1,909.00 (59.43%)	
Yes	4,062.00 (35.53%)	943.00 (30.59%)	1,014.00 (34.99%)	1,015.00 (35.97%)	1,090.00 (40.57%)	
**Smoke**, ***n*** **(%)**						**<0.001**
Never	7,152.00 (53.57%)	1,951.00 (50.95%)	1,848.00 (54.95%)	1,745.00 (53.64%)	1,608.00 (54.73%)	
Former	3,316.00 (26.36%)	827.00 (24.50%)	843.00 (26.02%)	826.00 (27.24%)	820.00 (27.67%)	
Current	2,587.00 (20.08%)	806.00 (24.56%)	634.00 (19.02%)	576.00 (19.13%)	571.00 (17.59%)	
**Educational**, ***n*** **(%)**						**<0.001**
≥High school	10,115.00 (85.24%)	2,612.00 (81.09%)	2,574.00 (84.84%)	2,509.00 (87.72%)	2,420.00 (87.30%)	
<High school	2,940.00 (14.76%)	972.00 (18.91%)	751.00 (15.16%)	638.00 (12.28%)	579.00 (12.70%)	
PIR	94.00 (1.00, 231.00)	96.00 (9.00, 225.00)	92.00 (1.00, 220.00)	94.00 (1.00, 233.00)	93.00 (1.00, 242.00)	0.4
**Hypertension**, ***n*** **(%)**						**<0.001**
No	8,302.00 (66.14%)	2,145.00 (61.95%)	2,080.00 (65.80%)	2,043.00 (67.66%)	2,034.00 (69.16%)	
Yes	4,753.00 (33.86%)	1,439.00 (38.05%)	1,245.00 (34.20%)	1,104.00 (32.34%)	965.00 (30.84%)	
**Diabetes**, ***n*** **(%)**						**<0.001**
No	10,729.00 (86.40%)	2,871.00 (84.54%)	2,674.00 (85.12%)	2,634.00 (87.74%)	2,550.00 (88.21%)	
Yes	2,326.00 (13.60%)	713.00 (15.46%)	651.00 (14.88%)	513.00 (12.26%)	449.00 (11.79%)	
TC, mg/dL	53.00 (25.00, 91.00)	56.00 (25.00, 93.00)	57.00 (28.00, 95.00)	50.00 (24.00, 89.00)	50.00 (22.00, 86.00)	**0.003**
HDL-C, mg/dL	19.00 (9.00, 33.00)	21.00 (10.00, 36.00)	19.00 (9.00, 32.00)	19.00 (10.00, 33.00)	18.00 (8.00, 32.00)	**<0.001**
LDL-C, mg/dL	46.00 (21.00, 79.00)	46.00 (22.00, 79.00)	48.00 (22.00, 81.00)	44.00 (22.00, 77.00)	44.00 (20.00, 76.00)	**0.020**
WBC, × 109/L	22.00 (9.00, 40.00)	24.00 (11.00, 43.00)	23.00 (10.00, 41.00)	21.00 (9.00, 38.00)	21.00 (9.00, 38.00)	**<0.001**
RBC, × 109/L	59.00 (26.00, 106.00)	57.00 (24.00, 106.00)	60.00 (25.00, 107.00)	60.00 (26.00, 105.00)	61.00 (28.00, 106.00)	0.6
PLT, × 106/L	77.00 (36.00, 135.00)	81.00 (37.00, 140.00)	77.00 (36.00, 134.00)	76.00 (35.00, 136.00)	73.00 (34.00, 131.00)	**0.044**
**Stroke**, ***n*** **(%)**						**<0.001**
No	12,556.00 (96.90%)	3,395.00 (95.37%)	3,189.00 (96.79%)	3,043.00 (97.25%)	2,929.00 (98.17%)	
Yes	499.00 (3.10%)	189.00 (4.63%)	136.00 (3.21%)	104.00 (2.75%)	70.00 (1.83%)	

a Median (IQR) for continuous; *n* (%) for categorical.

b Design-based Kruskal Wallis test; Pearson's X^∧^2: Rao & Scott adjustment. CI, confidence interval; BMI, body mass index; PIR Ratio of family income to poverty; TG, triglycerides; LDL-C, low-density lipoprotein cholesterol; HDL-C, high-density lipoprotein cholesterol; RBC, red blood cells; WBC, white blood cells; PLT, platelets. Values in bold are statistically significant (*p* < 0.05).

### 3.2 Association between the vitamin B1 and stroke

A weighted multiple logistic regression analysis was conducted to examine the relationship between vitamin B1 and stroke, considering variables such as age, sex, race, educational level, smoking, alcohol consumption, hypertension, and diabetes. Our findings indicate that vitamin B1 intake was negatively associated with the risk of stroke both before (OR: 0.64; 95% CI: 0.53–0.76) and after (OR: 0.84; 95% CI: 0.71–1.00) adjusting for covariates. Furthermore, participants were evenly divided into quartiles based on their vitamin B1 intake, and the results demonstrated that individuals with higher vitamin B1 intake had a lower risk of stroke both prior to and following the adjustment for covariates (Table 3).

**Table 3:** Weighted logistic regression analysis of vitamin B1 and stroke.

	**Non-adjusted model**		**Model I**		**Model II**	
	**OR (95% CI)**	***P*-value**	**OR (95% CI)**	***P*-value**	**OR (95% CI)**	***P*-value**
Vitamin B1	0.64 (0.53, 0.76)	<0.001	0.77 (0.64, 0.93)	0.007	0.84 (0.71, 1.00)	0.048
**Quartile**						
Q1	Ref.		Ref.		Ref.	
Q2	0.68 (0.48, 0.97)	0.035	0.72 (0.50, 1.04)	0.081	0.79 (0.53, 1.17)	0.210
Q3	0.58 (0.40, 0.86)	0.007	0.68 (0.46, 1.01)	0.056	0.79 (0.53, 1.16)	0.200
Q4	0.38 (0.28, 0.53)	<0.001	0.55 (0.39, 0.78)	<0.001	0.66 (0.46, 0.94)	0.022
*P* for trend	<0.001		<0.002		0.033	

Data are expressed as OR (95% CI). Model I was adjusted for age, gender and race/ethnicity. Model II adjusted for age, sex, race/ethnicity, education level, smoking status, alcohol consumption, BMI, diabetes, and hypertension.

**Figure 2:**
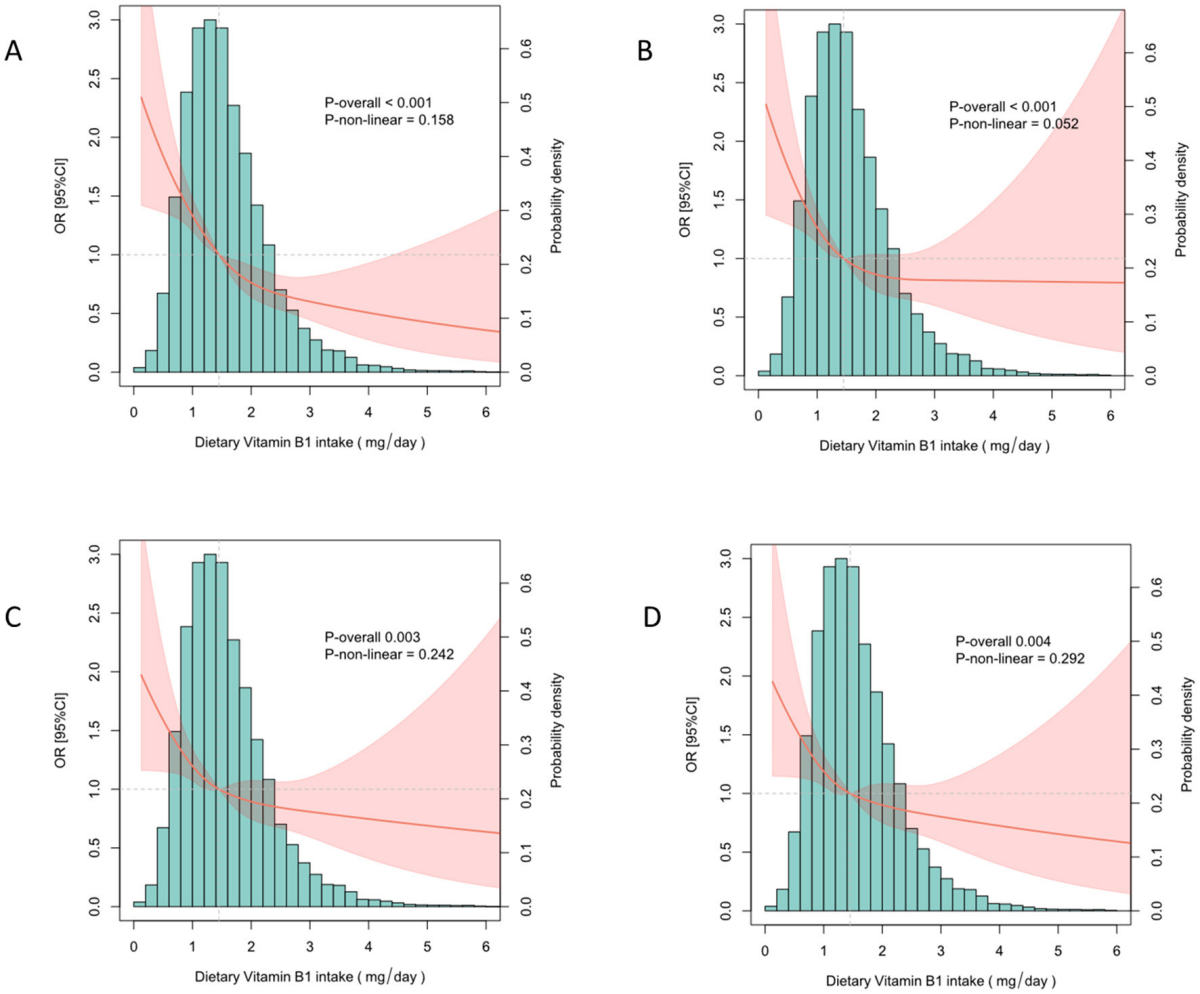
Analysis of the nonlinear relationship between vitamin B1 and stroke risk. **(A)** No adjustments made. **(B)** Adjusted for age, gender and ethnicity. **(C)** Adjusted for age, sex, race, education level, smoking, alcohol use, high blood pressure, diabetes. **(D)** Adjusted for all covariates.

Through RCS analysis, we identified a negative linear relationship between vitamin B1 intake and stroke risk in the unadjusted model (Figure 2A), Model I (Figure 2B), and Model II (Figure 2C) (*P* < 0.05 for linear trend). This linear relationship remained consistent when all variables were included in the analysis (Figure 2D), demonstrating the stability of this association. Prior to the inflection point (1.42 mg) of the negative linear relationship between vitamin B1 intake and stroke risk, there was a significant decreasing trend in stroke risk with increasing vitamin B1 intake. However, after the inflection point, the rate of decrease in stroke risk slowed with increasing niacin intake (Figure 2). Additionally, we found that this relationship persisted when the number of knots was set to 3 (Supplementary Figure S1).

**Figure 3:**
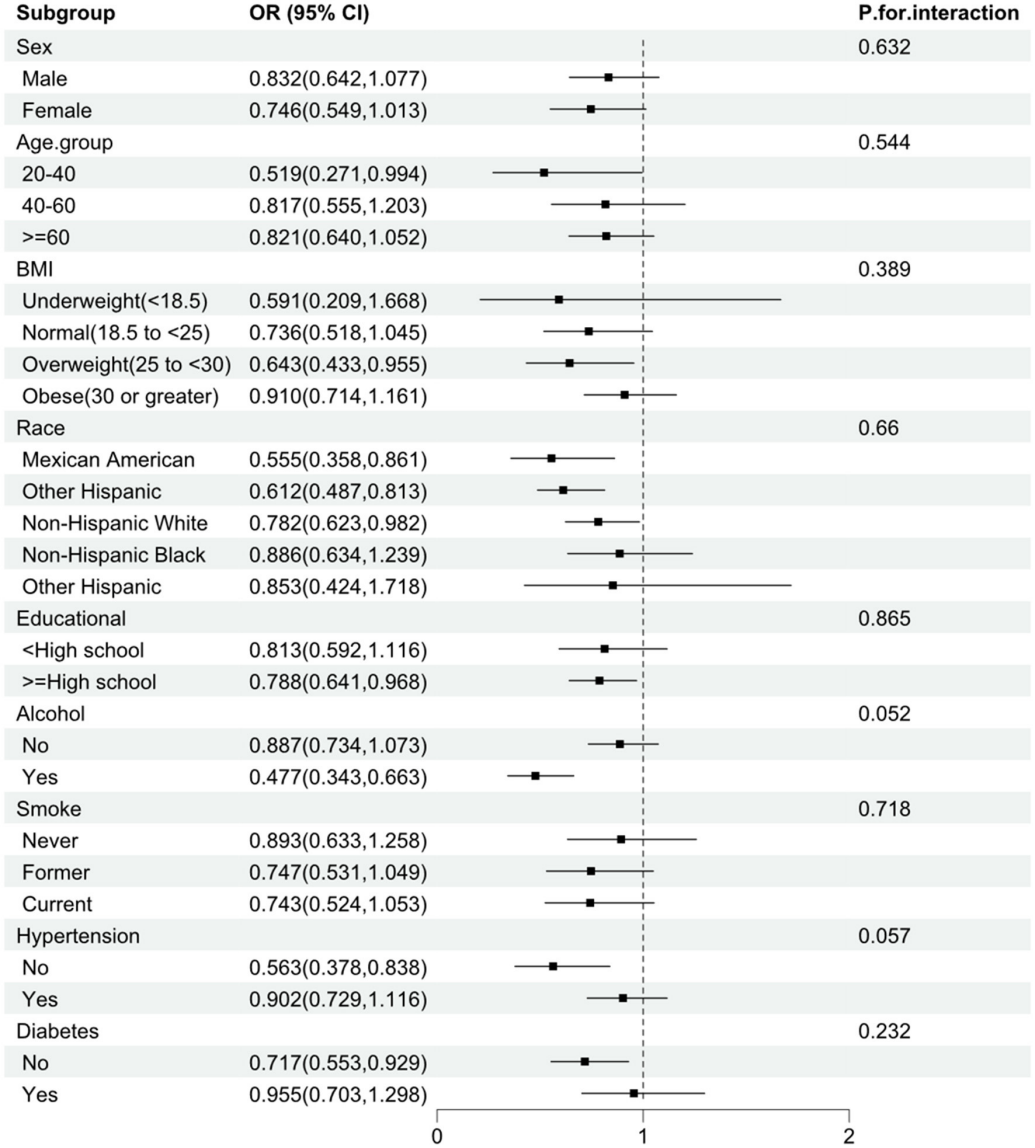
Subgroup analysis of the association between vitamin B1 and stroke. The analysis was stratified by sex, age, race, BMI, smoking, alcohol use, hypertension, and diabetes. Logistic regression analysis adjusted for age, sex, race, education, smoking, alcohol use, hypertension, and diabetes.

As shown in Figure 3, none of the stratified variables—including gender, age, race/ethnicity, BMI, smoking status, alcohol consumption, diabetes, and hypertension—significantly affected the association between vitamin B1 and stroke (all *P*-values for interaction > 0.05).

### 3.3 Development and validation of machine learning predictive models

**Figure 4:**
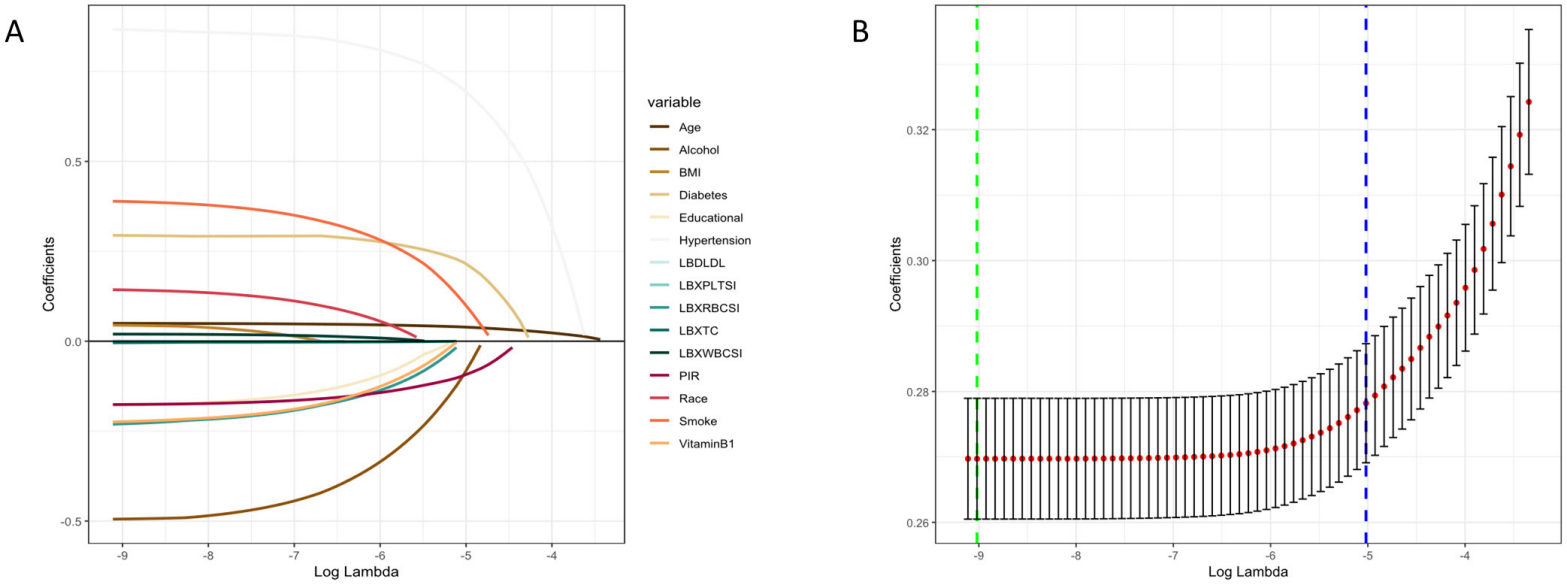
LASSO penalized regression analysis was used to identify factors associated with stroke risk. **(A)** The coefficient trajectory for each variable as Log Lambda changes. **(B)** Cross Validation Error (CVM) plot for different Log Lambda values, green dashed line represents minimum error (Log Lambda _ min), blue dashed line represents a standard error threshold selected by the model (Log Lambda_1se).

**Figure 5:**
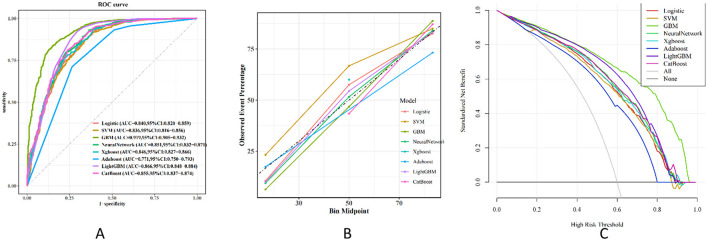
Performance and comparison of 8 different prediction models. **(A)** ROC curve. **(B)** Calibration curve. **(C)** Decision curve.

Due to the imbalance of binary classification labels in the initial dataset, SMOTE was employed to improve the performance of the machine learning (ML) model. By utilizing the SMOTE method, we synthesized samples to achieve a 1:1 class balance ratio in the final processed training set. A standard scale was determined for the selected features, and the data was randomly split into training and testing sets in a 7:3 ratio. Eight ML algorithms were developed in the training set to predict the risk of stroke occurrence. The algorithms used in this study applied the constructed models to the testing set. In this study, we employed univariate analysis to screen relevant variables, which were subsequently incorporated into LASSO regression for the selection of final feature variables (Figure 4). The lambda.1se parameter was chosen, resulting in the identification of seven variables: Diabetes, Hypertension, Smoking, Alcohol consumption, PIR, Age, and Vitamin B1. These selected clinical features were utilized to construct a stroke risk prediction model. The model's performance was evaluated using Decision Curve Analysis (DCA), Calibration Curve (CC), and the Area Under the Receiver Operating Characteristic (ROC) Curve (AUC) (Figure 5). The model was evaluated using accuracy, AUC, precision, recall, and F1 score (Table 4). We used 10-fold cross-validation to assess the model's performance, and the results showed that the GBM model achieved an accuracy of 84.2% and an AUC of 91.9%. The model's performance was compared with traditional methods, and the results indicated that it has superior predictive capability. The calibration curve is closely aligned with the diagonal, suggesting that the model is well-calibrated and does not exhibit significant overfitting. Consequently, GBM was selected for the subsequent phase of analysis.

**Table 4:** Performance comparison of eight machine learning (ML) models.

**Model**	**AUC**	**Accuracy**	**Sensitivity**	**Specificity**	**F1**
Logistic	0.840	0.787	0.863	0.674	0.83
SVM	0.836	0.787	0.873	0.659	0.831
GBM	0.919	0.842	0.837	0.85	0.864
Neural Network	0.851	0.787	0.798	0.771	0.818
Xgboost	0.846	0.775	0.783	0.763	0.807
Adaboost	0.771	0.721	0.711	0.736	0.754
LightGBM	0.866	0.835	0.924	0.703	0.871
CatBoost	0.855	0.792	0.812	0.761	0.824

**Figure 6:**
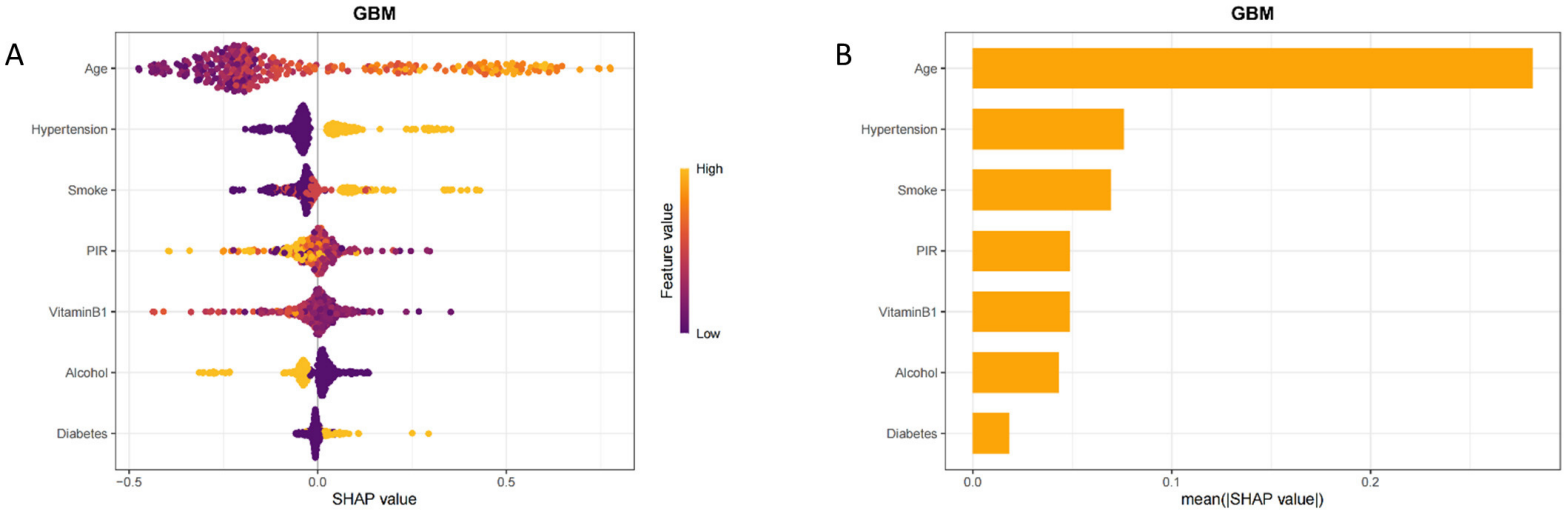
Interpretability analysis of the model. **(A)** SHAP tree diagram of GBM model characteristics. **(B)** Ranking of importance of GBM model features.

**Figure 7:**
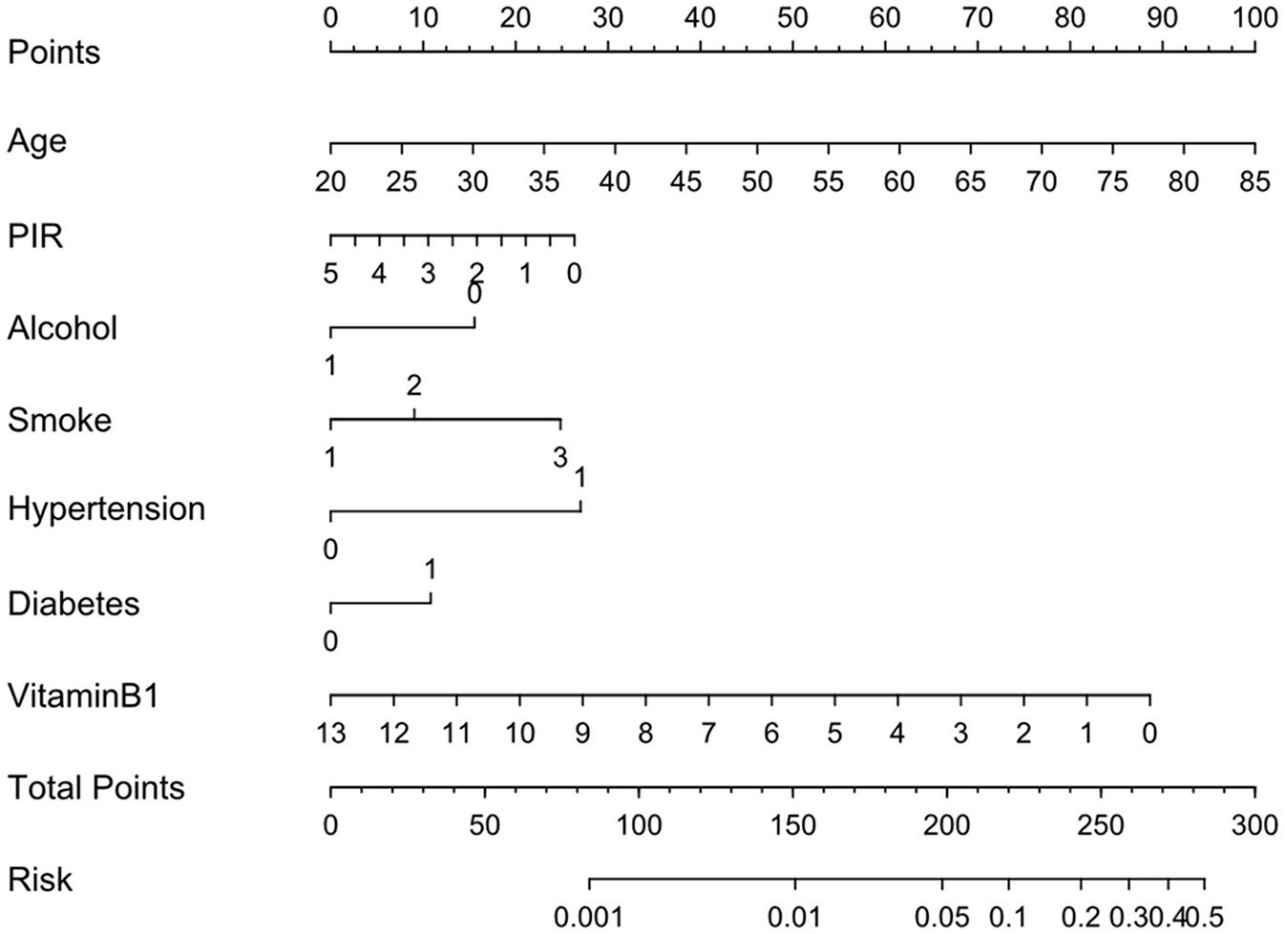
Nomograms from multiple logistic regression models for stroke risk prediction.

To enhance the model's interpretability, we used SHAP values to analyze the contribution of each feature to the model's output. The results showed that the vitamin B1 feature has a significant impact on the prediction (Figure 6). Individuals with high values primarily provide a negative contribution, while those with low values contribute positively. Given the commendable performance of the traditional logistic regression model in the preliminary analysis, we then proceeded to develop a nomogram based on the seven identified risk factors. By incorporating these seven risk factors, the nomogram facilitates a more precise estimation of the likelihood of specific outcomes (Figure 7).

## 4 Discussion

In this study, we conducted a cross-sectional analysis to investigate the relationship between dietary vitamin B1 intake and the risk of stroke. Our findings revealed a negative linear correlation between vitamin B1 intake and stroke incidence, which remained consistent even after adjusting for covariates. Subsequent subgroup analyses further confirmed the stability of this correlation across diverse groups. We conducted a secondary analysis using interview data collected by trained CDC personnel as part of NHANES, and employed univariate analysis alongside the LASSO regression model to screen pertinent clinical variables. The identified significant variables were utilized to develop predictive models using eight machine learning methods, including Logistic Regression (LR), Gradient Boosting Machine (GBM), XGBoost, Support Vector Machine (SVM), Neural Networks (NNET), AdaBoost, LightGBM, and CatBoost. This multi-model comparative validation underscored the importance of various key predictive factors and provided a valuable auxiliary tool for clinical decision-making.

Stroke is the second leading cause of death worldwide. According to the Global Burden of Disease Study (GBD), while the prevalence of stroke has decreased, the age, gender, and geographical distribution of patients suggest that the socioeconomic burden of stroke has risen over time (21). The pathological mechanism is closely associated with oxidative stress, endothelial dysfunction, vascular wall damage, and platelet activation and aggregation, ultimately resulting in intravascular thrombosis (22). The risk of stroke increases with age, doubling for both men and women after the age of 55 (23). Individuals suffering from conditions such as hypertension (21, 24, 25), coronary artery disease (26), or hyperlipidemia (27) face an increased risk of stroke. Notably, nearly 60% of stroke patients have a history of transient ischemic attack (TIA). While some stroke risk factors are immutable, others can be modified.

Vitamin B1, also known as thiamine, is an essential water-soluble vitamin for the human body, primarily involved in energy metabolism, the maintenance of nervous system health, and the regulation of cardiovascular functions (28–30). The earliest recorded accounts of thiamine deficiency can be traced back to the 3rd century; nonetheless, it was during the 19th century that the condition became notably more common, especially among sailors whose diets were limited to repetitive and bland foods. Researchers proposed that beriberi resulted from inadequate nutrition. It was put forward that the consumption of rice bran, which is usually eliminated in the process of converting brown rice into white rice, might help in avoiding this illness. Many efforts were undertaken to isolate and characterize specific chemical components found in rice bran, culminating in the discovery of the “anti-beriberi compound.” The definitive structure of this compound was established in 1936 and given the name thiamine. Hence, ensuring the inclusion of whole grains or fortified processed foods to avert thiamine deficiency is crucial. Thiamine significantly promotes cellular energy metabolism and, as a crucial cofactor in carbohydrate conversion, aids in providing energy to nerve cells, thereby maintaining their normal morphology and function (31). Furthermore, vitamin B1 indirectly participates in the synthesis of energy-consuming nucleic acids, neurotransmitters, and myelin sheaths. It also contributes to the speed of nerve conduction by maintaining myelin sheaths (32). Thiamine exerts a protective effect on the proliferation of human arterial smooth muscle cells mediated by glucose and insulin, which further prevents the occurrence of vascular atherosclerosis and reduces the risk of stroke (33). Thiamine functions as thiamine triphosphate (TTP) in neural membrane activity. It generates reducing power during glucose metabolism, thereby protecting cells from the damaging effects of oxidative stress and consequently safeguarding endothelial and vascular wall cells from oxidative stress injury. This mechanism is likely a key factor in its association with a reduced incidence of stroke. In a retrospective study (34), we found that vitamin B1 deficiency negatively impacts the rehabilitation treatment of stroke patients. However, the role of thiamine in the pathogenesis of stroke requires further exploration.

The complex of B vitamins consists of eight distinct vitamins (B1, B2, B3, B5, B6, B7, B9, and B12) that, although they do not share chemical similarities, are categorized together mainly because of their common coenzyme activities. These B vitamins are vital for numerous physiological processes in the human body and also have specific roles related to the nervous system. Commonly known as “neurotrophic” B vitamins, they are essential for the proper functioning of both the central nervous system (CNS) and the peripheral nervous system (PNS). In previous studies, we demonstrated that supplementation with B vitamins—specifically, vitamin B9 (folic acid), vitamin B12, and vitamin B6—can significantly reduce the overall relative risk of stroke by lowering the total homocysteine (tHcy) concentration in the blood (35). Despite some preliminary findings, there is currently a lack of literature reporting the effects of vitamin B1 on stroke risk. Our research indicates a significant linear negative correlation between dietary vitamin B1 intake and stroke incidence, suggesting that increasing dietary vitamin B1 may positively influence the reduction of stroke risk. However, this association, along with its specific mechanisms, requires further investigation. It is noteworthy that mammals cannot synthesize B vitamins independently and must obtain adequate amounts through their diet, highlighting the importance of dietary interventions.

In this study, we utilized national data combined with sample weights to assess the correlation between dietary vitamin B1 intake and the occurrence of stroke, thereby enhancing the generalizability of our findings to the U.S. population. The regression analysis, adjusted for covariates, along with the large sample size, enabled us to perform subgroup analyses that confirmed the robustness of our results. Furthermore, by comparing eight machine learning algorithms, we constructed a predictive model for stroke prevalence, ultimately identifying the most effective model. This model offers a practical approach for predicting stroke in individuals, thereby facilitating the development of targeted prevention and intervention strategies. Although our model performed well on this dataset, its generalizability may be somewhat limited due to the size and diversity of the dataset.

However, this study has specific limitations. Firstly, it is a cross-sectional study, which precludes causal inferences. Further prospective research is warranted to validate these findings. Secondly, the majority of the predictors utilized in our study were derived from self-reported data, which may introduce bias. Lastly, we conducted internal validation solely on the study dataset and lack an external cohort for further evaluation of the model's performance. Moreover, the sample was sourced from a single center, potentially limiting the generalizability of the research findings. Future studies should consider incorporating prospective designs and multi-center data, alongside integrating a broader range of patient data and employing advanced machine learning techniques. This approach would enhance the robustness and generalizability of the findings and ultimately contribute to the development of more personalized and precise treatment management strategies for stroke patients.

## 5 Conclusion

Our research indicates a significant linear negative correlation between dietary vitamin B1 intake and the prevalence of stroke among American adults. The machine learning model based on the Gradient Boosting Machine (GBM) method demonstrates strong predictive performance. However, further studies are needed to validate these findings, explore the underlying mechanisms, and assess the therapeutic potential.

## Data Availability

The original contributions presented in the study are included in the article/Supplementary material, further inquiries can be directed to the corresponding author.
