# Supplementary figures and images for: The relationship between dietary vitamin B1 and stroke: a machine learning analysis of NHANES data
Source: Front Nutr. 2025 May 6;12:1584654. doi: 10.3389/fnut.2025.1584654 (PMC12088975; doi:10.3389/fnut.2025.1584654)

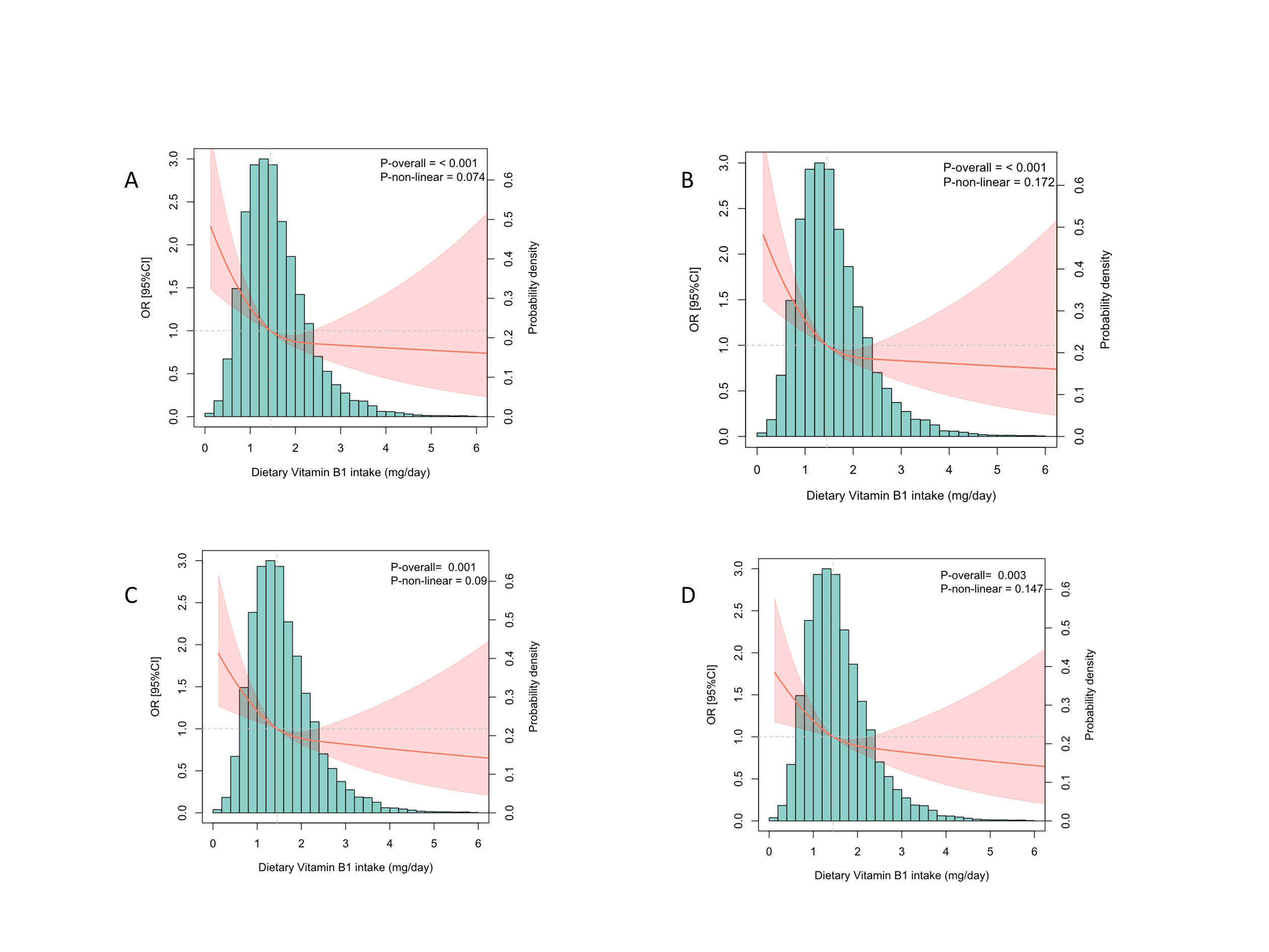

Supplement: Supplementary file 1 [file Image_1.tif]
